# Internal fights over resources: The effect of power struggles on team innovation

**DOI:** 10.3389/fpsyg.2022.996737

**Published:** 2022-11-16

**Authors:** Sung Mo Kang

**Affiliations:** Economics and Business Department, Cornell College, Mount Vernon, IA, United States

**Keywords:** power struggles, team innovation, social information processing theory, team learning, power hierarchy

## Abstract

Power hierarchy is a recently growing topic among scholars. Although the previous literature has emphasized the importance of understanding power hierarchy in teams and demonstrated the negative consequences of power struggles among team members in team performance, it neglected to explore how power struggles impact other team functioning and outcomes. Drawing on social information processing theory and the team learning behavior model discussed, this study proposes that power struggles send aggressive social information to team members, and such social information negatively influences team learning. Social information emitted by power struggles undermines psychological safety and creates hostility and interpersonal tensions, which reduce team members’ providing new ideas and information sharing. In addition, this study proposes a positive relationship between team learning and team innovation since team learning provides two key conditions (i.e., active knowledge integration and appropriate team climate) for successful team innovation. Lastly, this study suggests the mediating role of team learning between power struggles and team innovation. Using a sample of 99 teams from two organizations in Korea, this study tested the proposed model. In sum, this study found that (1) power struggles are negatively related to team learning, (2) team learning is positively related to team innovation, and (3) team learning mediates the relationship between power struggles and team learning.

## Introduction

Relationships among team members are not always harmonious but often conflictual (Jehn, 1995; Tekleab et al., 2009; Greer et al., 2011). Team members often compete with each other to gain valuable but finite resources within a team, such as pay, incentives, personnel, or information (Magee and Galinsky, 2008). Due to such features of organizational resources (i.e., valuable and limited), how resources are equally or unequally distributed within a team or who gains more or less are important issues among team members (Greer and Van Kleef, 2010; Kilduff et al., 2016; Greer et al., 2017). In addition, individuals inherently desire and pursue higher power within a group (Magee and Galinsky, 2008; Lammers et al., 2016). Thus, team members often engage in specific behaviors, called power struggles, in order to gain more resources than others and increase their resource controllability within a team (Greer and Van Kleef, 2010; Bendersky and Hays, 2012; Van Bunderen et al., 2018).

Power struggles are defined as the competition among team members over valuable but limited organizational resources and their controllability (Greer and Van Kleef, 2010). Despite the substantial body of power struggles on teams, previous literature has mostly focused on hampered team performance as a consequence of power struggles (e.g., Greer and Van Kleef, 2010; Bendersky and Hays, 2012; Greer et al., 2017; Van Bunderen et al., 2018). Indeed, scholars neglected to understand why and how power struggles impair other team functioning or outcomes with different mechanisms.

To address this unexplored area, this study grounds social information processing theory (Salancik and Pfeffer, 1978) in the team learning behavior model posed by Edmondson (1999) to explore the effect of power struggles on team innovation through team learning. Here, team learning refers to a process of communicating knowledge, seeking feedback, and discussing outcomes (Edmondson, 1999), and team innovation refers to the introduction and application of novel ideas (West and Farr, 1990). Social information processing theory argues that social information in the workplace affects employees’ judgments and evaluations of their work environment, which makes employees adjust their attitudes and behaviors. Based on the integration of social information processing theory and the team learning behavior model, I propose that power struggles within a team trigger perceptions and beliefs that other team members are not coworkers, but competitors who can threaten an individual’s resources and its controllability (Greer et al., 2017). Such perceptions triggered by power struggles then may undermine team psychological safety and create a hostile atmosphere within teams, hindering team learning. As a result, reduced team learning may make team members not be free to exchange information or feedback, express new or experimental ideas, and make decisions collaboratively, harming team innovation.

This study intends to make the following contributions. First, this study contributes to social information processing theory. More specifically, this study contributes to understanding how power dynamics among team members create a social environment sending social cues to team members, which impacts team processes and outcomes. Second, this study contributes to the literature on power hierarchy, which lacks both theoretical and empirical works investigating the potential mediator and its consequence of power struggles (e.g., Greer and Van Kleef, 2010; Van Bunderen et al., 2018; for exceptions, see Greer and Chu, 2020). More specifically, this study contributes to understanding the additional pathway of the effect of power struggles on teams and how and why the effect takes place. Last, this study contributes to the team learning behavior model (Edmondson, 1999; Van den Bossche et al., 2006). This study extends the framework of the team learning behavior model by examining that power dynamics within a team may both support and impede team learning. Previous literature on the team learning behavior model has mainly investigated the antecedent conditions of team learning (e.g., Edmondson, 1999; Van Woerkom and Van Engen, 2009; Koeslag-Kreunen et al., 2018), but the understanding of how conflicts from competition over resources (i.e., power struggles) function still does not exist. Thus, this study expands an existing research framework on the team learning behavior model.

## Literature review and theory development

### Social information processing theory

According to social information processing theory (Salancik and Pfeffer, 1978), employees do not work in a vacuum; social contexts of the work environment influence employees to adapt their work-related attitudes, behaviors, and beliefs. In other words, individuals process the social information in the workplace and adjust their behaviors by observing, interpreting, and learning from social cues emitted by work environments or others. Previous literature has already demonstrated various types of social information sources and their effect on employees, such as work climate (e.g., Mawritz et al., 2012) and supervisors (e.g., Kang, 2022). For example, Lu et al. (2019) have empirically demonstrated that servant leadership sends social cues that leaders care about team members’ feelings and well-being, consequently improving the level of trust in leaders and the quality of emotional labor.

In the context of teams, social contexts of the work environment send important social information to all team members, and perception and interpretation of such social information influence how team members work together and interact with each other. This study assumes that the social information processing theory can serve as a suitable research framework to explore the effect of power struggles on team processes and outcomes. This reasoning is based on the fact that team members are important sources of social information to influence team members because team members interact with each other on a daily basis, easily observe and learn from others’ behaviors, and depend on each other for shared goals and objectives (Campion et al., 1993; Wageman, 1995; Hu and Liden, 2013). Thus, this study argues that power struggles among team members send specific social information to all team members, and perception and interpretation of such social information are circulated within the entire team, consequently influencing the interaction of team members and team processes.

### Power struggles and team learning

Power is defined as the ability to control valuable but limited resources (Keltner et al., 2003; Greer and Van Kleef, 2010). Multiple factors, such as job titles, skills, ability, and expertise are bases of hierarchical differentiation, often resulting in some possessing higher power than others within a team (Magee and Galinsky, 2008). Thus, high-power individuals have higher resource controllability than low-power individuals. However, individuals inherently desire and pursue higher social standings and power (Anderson et al., 2001). In addition, power is often seen as zero-sum, indicating that one member’s behavior seeking power can threaten others within a team (Magee and Galinsky, 2008; Greer et al., 2017). For these reasons, team members often engage in specific behaviors to gain more power than others, maintain the current relative level, or challenge other members’ power.

Such behaviors are called power struggles, defined as the extent to which team members compete over the relative levels of resources within a team (Greer and Van Kleef, 2010). Power struggles occur with a desire to change the relative levels of resource allocation among team members. They include a variety of behaviors, such as forming coercion, undermining others’ contributions (or exaggerating one’s contribution), criticizing others’ proposals, spreading gossip, or engaging in political behaviors (Greer et al., 2017). Power struggles can be directed upward from lower-ranked members to higher-ranked in order to bring them up, or vice versa (Greer and Van Kleef, 2010). For example, higher-ranked individuals sideline or undermine lower-ranked members to maintain their current level of power within a team. Still, lower-ranked members can intentionally withhold important information or spread gossip about higher-ranked members to put higher-ranked members down. As we see, forms of power struggles can be either overt or covert in all directions. The negative effect of power struggles on team performance has long been acknowledged (for reviews and meta-analytic evidence, see Greer et al., 2017, 2018).

In this study, I argue that power struggles are closely related to team learning beyond their effects on team performance. Here, team learning is defined as “an ongoing process of reflection and action, characterized by asking questions, seeking feedback, experimenting, reflecting on results, and discussing errors or unexpected outcomes of actions.” (Edmondson, 1999, p. 353). Researchers develop a conceptualization of team learning with two different approaches by considering team learning as a team outcome from learning (e.g., Ellis et al., 2003; Wilson et al., 2007) or as a process (e.g., Edmondson, 1999; Van den Bossche et al., 2006). In this study, I focus on learning process, not outcome, because power struggles send a signal and consequently change interaction among team members, and such changes in team members’ interactions influence how team learning is processed.

The team learning behavior model posed by Edmondson (1999) has demonstrated that team members’ shared perception or belief about their team plays an important role in the team learning process. This model has been implicitly based on Input-Process-Output (IPO) framework for studying teams (McGrath, 1984) with shared beliefs and perceptions about the team as input (e.g., team psychologically safety and group potency), team learning as a process and learning outcomes as an output (Knapp, 2010). For example, Edmondson (1999) and Van den Bossche et al. (2006) empirically tested and confirmed the effect of shared perception (e.g., psychological safety) as input on the team learning process and the mediation effect of team learning as a process for the relationship between shared beliefs of teams and team effectiveness.

Team learning includes dynamic communication and facilitation processes among team members, leading to changes and improvement (Decuyper et al., 2010). Based on the team learning behavior model (Edmondson, 1999), effective team learning processes include various behaviors, such as seeking feedback, experimenting, reflecting on results or errors, collaborative problem-solving, and information and knowledge sharing (Edmondson, 1999; Decuyper et al., 2010). In order for team learning to be successful (Edmondson, 1999), team members need to be free to (a) express new or experimental ideas without being scared of mistakes or rejection from other team members, (b) have opportunities to freely exchange feedback and constructively discuss errors or results, and (c) make decisions collaboratively with shared goals/visions by openly sharing knowledge and information.

I argue that power struggles within a team send certain social information to others. It prompts team members to view their team members as individuals who compete over limited valuable resources, try to change the current distribution of resources, and consequently threaten their resource controllability (Greer et al., 2017). Team members in high power struggle teams perceive such social information as aggressive and are more likely to believe that all team members seek their own beneficial resources and controllability for themselves at the cost of others’ power. These perceptions can be interpreted as threatening since power is valuable but limited (i.e., zero-sum game), and such perceptions are easily circulated within the entire team (Greer et al., 2017). Aggressive cues caused by power struggles, in turn, decrease the likelihood of team learning for the following reasons.

First, aggressive cues by power struggles create a psychologically unsafe working environment. Power struggles are contagious, spreading quickly throughout the entire team (Greer et al., 2017). Once triggered by one or more members, power struggles rapidly cause the whole team to be involved since individuals are sensitive to losing power and control (Magee and Galinsky, 2008). In a working environment where team members believe others are competitors (i.e., once power struggles are triggered; Greer et al., 2017), team members psychologically feel unsafe (De Hoogh et al., 2015; Lee et al., 2018). In a psychologically unsafe environment, team members are hesitant to provide new or experimental ideas (Edmondson, 1999). Team members in high power struggles may regard voicing new ideas or suggestions as risky since competitors can underestimate and ignore new ideas (Kilduff et al., 2016), resulting in low quality of the team learning process.

Second, social information emitted by power struggles creates hostility and tensions among team members. Once team members engage in power struggles, team members receive aggressive social cues and perceive tensions and hostility among team members (Greer et al., 2017). Interpersonal tensions and hostility within a team hinder intra-team trust (De Jong and Elfring, 2010). In a hostile and distrusting working environment, team members do not trust each other and stop sharing critical information and knowledge because distrust leads to decreased dialogue and shared communication for creating opportunities for knowledge sharing (O’Reilly et al., 1987). Previous literature has already demonstrated interpersonal tensions caused by power struggles hurt team information sharing (Greer and Van Kleef, 2010; Bendersky and Hays, 2012), which is a critical factor in team learning conditions (Edmondson, 1999). On the basis of the above arguments, I propose that

*Hypothesis 1*: Power struggles are negatively related to team learning.

### Team learning and team innovation

According to West and Farr (1990, p. 9), innovation is defined as “the intentional introduction and application within a role, group or organization of ideas, processes, products, or procedures, new to the relevant unit of adoption, designed to significantly benefit the individual, the group, organization or wider society.” As the business environment is changing quickly, and organizations are facing unexpected and unavoidable challenges such as the COVID-19 pandemic (Yuan et al., 2021), innovation is required for sustainable competitive advantage and survival (Leifer et al., 2000; Tushman et al., 2002). Since many organizations increasingly rely on teams (Ilgen et al., 2005), and team innovation benefits individuals, teams, and entire organizations, academic interests in team innovation have also been growing (see Van Knippenberg, 2017). Followed by definition of innovation of West and Farr (1990), team innovation can be defined as introducing and applying novel and useful ideas, processes, products, or procedures to the team. Although there is a conceptual overlap between team innovation and team creativity, they are not synonymous concepts. Team innovation includes both the generation of novel ideas, which is defined as team creativity, and the implementation of ideas into new products, processes, and procedures (West and Anderson, 1996; Amabile, 2000).

Review of team innovation of Van Knippenberg (2017) has suggested two critical conditions of team innovation: knowledge integration and team climate. First, in the knowledge integration perspective, previous literature has demonstrated that when team members bring different knowledge, information, and expertise to the team (e.g., Drach-Zahavy and Somech, 2001; Bell et al., 2011; Van Dijk et al., 2012) and actively share their knowledge with other team members (Jin and Sun, 2010; Hu et al., 2012; Kessel et al., 2012), team members can use the larger pool of information resources, and it helps the integration of different knowledge, information, and expertise for the realization of team innovation. This reasoning is based on the idea that different people know different things, and integrating such things (i.e., knowledge, information, and expertise) leads to novel and useful idea development, facilitating team innovation.

Second, from the team climate perspective, scholars have argued that team members’ shared perceptions are closely related to team innovation. For example, when team members have collective goals (e.g., Tjosvold et al., 2004) or feel participation safety within a team (all feel they can say anything they want; e.g., West, 1990), team members are more likely to be involved in the process of team innovation. In sum, team innovation is facilitated (1) when team members bring and share different information and knowledge within a team and (2) when team members have collective goals and feel psychologically safe within a team.

In line with the current study, I propose that team learning is positively related to team innovation based on the team learning behavior model (Edmondson, 1999) that argues the high level of team learning process (i.e., active knowledge and information sharing, frequent expression of experimental ideas, and collaborative decision making) are critical conditions of learning outcomes. In environments facilitating team learning: First, team members freely share information, knowledge, and expertise within a team, which facilitates team innovation. Second, team members feel free to express their new or experimental ideas, leading to higher team innovation. Third, team members make decisions collaboratively with shared goals, positively impacting team innovation. Given the theory and empirical evidence above, I propose that

*Hypothesis 2*: Team learning is positively related to team innovation.

Based on my prior arguments, I suggest that power struggles are likely to negatively influence team innovation through reduced team learning. Guided by social information processing theory (Salancik and Pfeffer, 1978) and the team learning behavior model (Edmondson, 1999), power struggles send social information to trigger team members to perceive that team members are competitors, not coworkers. Such social information is interpreted as aggressive cues, so it impacts how team members perceive their working environment and interact with others. Such aggressive cues hinder team learning because they create a psychologically unsafe working environment and hostility and tensions among team members, which make team members hesitate to provide new ideas and reduce knowledge and information sharing, and harm team communication. As a result, team innovation is hindered. In combination, the relationships predicted in Hypotheses 1 and 2 lead to the final step in my conceptual analysis: the prediction that team learning mediates the relationship between power struggles and team innovation.

*Hypothesis 3*: Team learning mediates the relationship between power struggles and team innovation such that the indirect effect is negative.

## Materials and methods

### Sample and procedure

Survey data were collected from full-time employees and supervisors within two industries, including academia and social welfare, to test the hypotheses articulated above. Employees with these organizations are grouped into teams, which range from 2 to 8 members. In the data collected, team members are responsible for a variety of tasks, such as general affairs, customer service, and business and product development. They have frequent interactions with each other on a daily base. In addition, team members have diversified knowledge and perspectives from various backgrounds and demographic characteristics.

This study used a multi-temporal and multi-source research design to avoid common method bias (Podsakoff et al., 2003, 2012). I tried to measure predictor and criterion variables at different points in time because measures of different constructs at the same point in time can produce artifactual covariance between team learning and team innovation. In addition, I tried to use two different sources (i.e., team members and team leaders) to reduce common rater bias because team members’ self-reported their innovative performance can be biased or overestimated (or underestimated). Specifically, I surveyed participants in two-time periods with 1-month time lag. At time 1, I asked employees to assess power struggles and team learning. At time 2 (1 month after Time 1), I asked team leaders to assess their team innovation. Thus, this study tried to diminish or eliminate the effect of common rater biases and contextual effects that may bias responses in order to gain more valid results.

This study used full information maximum likelihood estimation (FIML) for the treatment of missing data. Since the pattern of missing data indicated “missing completely at random (MCAR)” with a nonsignificant Little’s test [χ^2^(58) = 65.53, *p* = 0.23], and estimates with FIML are unbiased under MCAR (Newman, 2014), I chose FIML to deal with missing data. The final sample consisted of 387 employees and 99 supervisors. For the team member sample, 52% were women, and the average age was 36.22 years (SD = 9.15). For the team leader sample, 44% were women, and the average age was 46.86 years (SD = 8.71). The average organizational tenure was 6.75 years (SD = 3.99) for team members and 8.60 years (SD = 3.76) for team leaders.

### Measures

All measures were rated on a scale with scores ranging from 1, “strongly disagree,” to 5, “strongly agree.” Because all measures were originally constructed in English, I followed a double-blind translation-back translation procedure to ensure equivalence of meanings (Brislin, 1986) for making Korean versions of the survey instrument.

#### Power struggles

Power struggles were measured using a three-item scale developed by Greer and Van Kleef (2010). Items include (1) Team members try to dominate each other, (2) Team members argue about hierarchical order in team, and (3) Team members compete for control in the team. The reliability of power struggles was.92. Individual ratings of power struggles were aggregated to the mean level of that. The mean r_wg_ and ICCs values of power struggles were acceptable with an r_wg_ = 0.86, ICC(1) = 0.31 (*F* = 5.23, *p* < 0.001), and ICC(2) = 0.83. The results of this analysis legitimized the aggregation of team-level variables (James et al., 1984; Bliese, 2000; Klein and Kozlowski, 2000).

#### Team learning

Team learning was measured using a seven-item scale developed by Edmondson (1999). Sample items include (1) This team does not work without stopping to consider all the information team members have, (2) This team regularly takes time to figure out ways to improve its work performance, (3) This team actively reviews its own progress and performance, (4) This team ignores feedback from others, (5) This team asks for help from others in the company when something comes up that team members do not know how to handle, (6) This team relies on outdated information or ideas, and (7) This team asks others for feedback on its performance. The reliability of team learning was 0.80. Individual ratings of team learning were aggregated to the mean level of that. The mean r_wg_ and ICCs values of team learning were acceptable with an r_wg_ = 0.80, ICC(1) = 0.28 (*F* = 3.27, *p* < 0.001), and ICC(2) = 0.71, indicating acceptable group mean reliability and suggesting support for the aggregation of ratings to the team level (James et al., 1984; Bliese, 2000; Klein and Kozlowski, 2000).

#### Team innovation

Team innovation was measured using a four-item scale adapted by De Dreu and West (2001). Sample items include (1) Team members often implement new ideas to improve the quality of our products and services, (2) This team gives little consideration to new and alternative methods and procedures for doing their work, (3) Team members often produce new services, methods or procedures, and (4) This is an innovative team. The reliability of team innovation was 96.

### Data analysis

Since the teams were collected from various organizations, controlling for team-related factors is important for valid results (Johns, 2006). First, I controlled for team size since team size can affect team dynamics and various outcomes (e.g., Lee and Farh, 2004; Aubé et al., 2011). Further, I also controlled team-level organizational tenure since previous literature consistently has shown that longer-tenured employees have more job-related knowledge and better performance (Ng and Feldman, 2010), which may affect team learning and innovation.

Prior to hypothesis testing, I conducted descriptive, correlation, reliability, and confirmatory factor analysis (CFA). To test the proposed theoretical model, I conducted structural equation modeling (SEM) with the maximum-likelihood algorithm using MPLUS 8.7 (Muthén and Muthén, 1998–2017). Through SEM, I examined the path coefficients, their significant levels, and fit indices (chi-square, CFI, TLI, and RMSEA). In addition, I used a bootstrap approach to test the indirect effect by obtaining a confidence interval with 5,000 bootstrap samples. Bootstrapping is recommended for mediation analysis since it does not assume the normal distribution of indirect effects (Preacher and Hayes, 2004; Koopman et al., 2015). To assess the significance of the indirect effect, I check whether 95% CI does include 0 (the indirect effect is not statistically significant within 0.05 level) or not (the indirect effect is statistically significant within 0.05 level).

## Results

### Measurement model testing

To ensure the construct distinctiveness of measures, I employed CFA using MPlus 8.7 (Muthén and Muthén, 1998–2017). The result shows that the hypothesized three-factor model including power struggles, team learning, and team innovation has acceptable fit [χ^2^ (74) = 453.62, *p* < 0.01; CFI = 0.94; TLI = 0.92; RMSEA = 0.07; Hu and Bentler, 1999]. In addition, I compare the baseline three-factor model with alternative models with fewer factors to confirm that the hypothesized model is better than the alternatives. A two-factor model combining team learning and team innovation into one factor provided worse fit [χ^2^ (76) = 1613.24, *p* < 0.01; CFI = 0.83; TLI = 0.82; RMSEA = 0.12], compared to baseline three-factor model. In addition, I tested a single-factor model where all items are specified into a single latent variable, and the result showed that a single-factor model had a significantly worse fit [χ^2^ (77) = 2831.37, *p* < 0.01; CFI = 0.73; TLI = 0.72; RMSEA = 0.14]. The results of CFA are displayed in Table 1.

**Table 1:** Results of confirmatory factor analyses.

Model	*x* ^2^	df	Value of *p*	CFI	TLI	RMSEA
One-factor model	2831.37	77	*p* < 0.001	0.73	0.72	0.14
Two-factor model	1613.24	76	*p* < 0.001	0.83	0.82	0.12
Three-factor model	453.62	74	*p* < 0.001	0.94	0.92	0.07

Two-factor model: team learning and team innovation combined; One-factor model: power struggles, team learning, and team innovation combined.

### Descriptive statistics

Table 2 presents the means, standard deviations, Cronbach’s α, and intercorrelations for all variables. As I predicted, power struggles are negatively related to team learning and team innovation, and team learning is positively associated with team innovation.

**Table 2:** Descriptive statistics.

Variables	M	SD	1	2	3	4	5
1. Team size	3.91	1.84	–				
2. Team organizational-tenure	6.75	3.99	0.13	–			
3. Power struggles	2.06	1.04	−0.08	−0.14	(0.92)		
4. Team learning	3.54	0.59	−0.20	0.02	−0.64^**^	(0.80)	
5. Team innovation^a^	3.37	0.81	0.05	0.14	−0.55^**^	0.55^**^	(0.96)

^**^p < 0.01. ^a^Rating provided by team leaders. Figures on the diagonal are Cronbach’s α.

### Hypothesis testing

**Figure 1:**
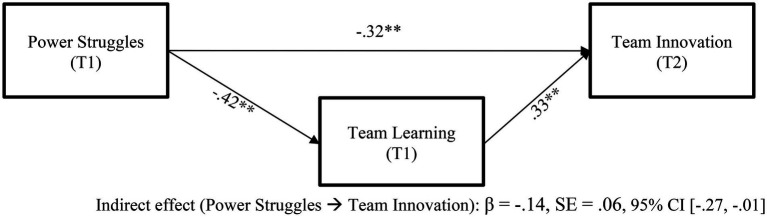
Summary of hypotheses. *p < 0.05. **p < 0.01.

The hypothesized structural model showed a good fit [χ^2^ (9) = 167.47, *p* < 0.01; CFI = 0.99; TLI = 0.98; RMSEA = < 0.01]. Hypothesis 1 predicted that power struggles would be negatively related to team learning, and Hypothesis 2 predicted that team learning would be positively related to team innovation. In support of Hypothesis 1, I found that power struggles negatively influence team learning with controlling effect on team size and team-organizational tenure (β = −0.42, SE = 0.03, *p* < 0.001). In support of Hypothesis 2, the results showed that team learning positively influences team innovation with controlling effect of team size and team-organizational tenure (β = 0.33, SE = 0.15, *p* = 0.03). Thus, Hypotheses 1 and 2 are supported. In addition, I predicted that team learning would mediate the relationship between power struggles and team innovation. I investigated mediation analysis to test Hypothesis 3 to test whether or not team learning mediates the relationship between power struggles and team innovation. As I did for Hypotheses 1 and 2, team size and team-organizational tenure were also controlled. The finding suggests that high power struggles tend to have lower team innovation through weakened team learning. The bootstrap results with 5,000 re-samples confirmed the significance of indirect effect, bootstrapping at a 95% confidence interval (β = −0.14, SE = 0.06, 95% CI = −0.27, −0.01). A full depiction of the results is shown in Figure 1.

## Discussion

Drawing on the integration of social information processing theory (Salancik and Pfeffer, 1978) and the team learning behavior model (Edmondson, 1999), I proposed that power struggles (a) provide important social information that triggers team members to perceive that other team members are competitors and (b) influence how team learning is processed. To explain how and why power struggles impact teams negatively, I proposed the team learning process as a critical transmitter of power struggles on teams. In particular, I hypothesized that team power struggles negatively impact team innovation through reduced team learning. I found strong support for the negative relationship between power struggles and team learning and the positive relationship between team learning and team innovation. In addition, this study also found the mediating effects of team learning between power struggles and team innovation. I discuss the implications of this study in more detail below.

### Theoretical implications

The theoretical implications of this study advance the existing literature 3-fold. This study contributes to social information processing theory (Salancik and Pfeffer, 1978). Although team members are a significant source of social information within a team, previous literature on social information processing mostly focused on leadership (e.g., Boekhorst, 2015; Qian et al., 2020) and team climate (e.g., Mayer et al., 2010; Mawritz et al., 2012). This study has demonstrated that behaviors of team members can signal important social cues to team members and influence team processes and outcomes. In this regard, this study expands our understanding of how team members provide social information to other team members, how such information is circulated within a team, and how it impacts the interaction/process of team members and team outcomes.

Second, this study expands the literature on power hierarchy. Although extant literature focuses on team performance following power struggles (e.g., Greer et al., 2017, 2018), I clearly demonstrate how power struggles influence other team outcomes, not team performance. This study provided empirical support that team learning is influenced by power struggles, subsequently impacting team innovation. This focus on power hierarchy contributes to our general understanding of how team members react to power hierarchy and how their reactions affect team outcomes. In sum, this study helps broaden our understanding of how power hierarchy impacts teams.

Third, we contribute to the existing theoretical framework in the team learning behavior model by suggesting an alternative antecedent: power struggles. Although previous literature has suggested various antecedents affecting team learning, such as team climate (Edmondson, 1999), team characteristics (Ellis et al., 2003), leadership (e.g., Koeslag-Kreunen et al., 2018), and different types of such as task and relational conflicts (e.g., Van Woerkom and Van Engen, 2009), how power hierarchy affects team learning is relatively understudied. Our study broadened our understanding of the role of power struggles within a team as an important antecedent of team learning by making team members psychologically unsafe and creating a hostile working environment. Following this, future scholars could consider how other variables relevant to power hierarchy (e.g., power dispersion and power structure) could influence team learning. In this regard, this study opens the door for scholars to consider the role of power hierarchy in team learning.

### Practical implications

First and foremost, this study’s findings indicate that power hierarchy plays a pivotal role in team innovation. It is, therefore, in the interest of organizations to enhance team innovation by lowering the level of power struggles within teams. First, to improve team learning, team leaders need to perceive and sense power distribution and dynamics within team members and mediate frictions or troubles caused by power struggles between team members. Team leaders should realize power struggles seriously hinder team learning, subsequently hampering team innovation, as discussed in this study. Second, as the previous literature discussed, power struggles mostly arise when power is unequally distributed (i.e., high power dispersion; Greer and Van Kleef, 2010) among team members. In highly power dispersed teams, team members are more likely to protect, attain, and maintain current power (e.g., Brett et al., 2007) or challenge other’s power (e.g., Polzer and Caruso, 2008), resulting in a high level of power struggles. Thus, team leaders are required to understand the negative consequences of internal fights over resources on teams, navigate and curb power struggles, and try to distribute power to all team members equally. At the same time, team members should also be aware that excessive competition over resources harms team learning and innovation, ultimately destroying the entire team and themselves.

Second, this study emphasizes the importance of team learning for team innovation. As I demonstrate, when team members actively engage in team learning behaviors, it improves team innovation. The previous literature has suggested many antecedents that organizations can provide to employees, such as team leader coaching, team climate, and training systems (Edmondson, 1999; Kozlowski and Bell, 2007). Thus, organizations need to develop and provide appropriate environments or training programs to increase team learning behaviors for improving team innovation and further organizational innovation.

### Limitations and suggestions for future research

Several limitations concerning this study and future directions should be noted. First, the data collection for this study was from South Korea, so it may limit the generalizability of the findings since contextual factors such as culture can have confounding effects on research findings (Johns, 2018). For example, with some cultural backgrounds, power struggles may stimulate employee’s learning behaviors and influence team innovation positively. Thus, future research may test the hypothesized model of this study in different countries with different cultural backgrounds.

Second, although I limited my exploration of a mediator to team learning in this study, future research should continue to investigate other mediators with different theories that can explain the relationship between power struggles and team innovation. For example, power struggles can negatively influence cognitive emergent states such as team cognition (Marks et al., 2001; DeChurch and Mesmer-Magnus, 2010), which positively influences a variety of team outcomes (Rapp et al., 2021).

Last, future research can consider boundary conditions of the theoretical model I suggested, such as leader characteristics and leadership styles. Team leader characteristics or leadership styles might alleviate or aggravate the effect of power struggles on teams. For example, managers with high political skills may be good at sensing power struggles and resolving conflicts (Munyon et al., 2015) caused by power struggles, buffering the negative effect of power struggles on team learning. As an additional example, De Hoogh et al. (2015) explored the autocratic leadership can amplify the negative effect of power struggles on team performance. Leader characteristics and leadership styles have long been studied as important contexts within teams, so there are still many opportunities for future research to consider the contextual effects of power struggles on team behavioral outputs.

Another important boundary condition is team developmental stages. Although this study considers team-level organizational tenure as a control variable, team development stages might play important boundary conditions of the suggested model. For example, a model of developmental stages for a team (Tuckman, 1965; Tuckman and Jensen, 1977) has suggested that teams in different stages show different aspects of team behavioral characteristics, intragroup conflicts, and interdependence. Depending on the developmental phases of team building, team members might react differently against power struggles, changing the extent of negative effects or even bringing positive effects on team learning and innovation. Thus, investigating a wider range of moderators would further extend our understanding of power struggles within team contexts.

## Conclusion

This study aimed to examine the negative effect of power struggles on team learning and innovation. Based on social information processing theory and the team learning behavior model, I found that power struggles negatively influence team learning, subsequently hindering team innovation. The results of this study highlight the importance of an additional mediator and mechanism for explaining the negative consequences of power struggles within teams.

## Data availability statement

The raw data supporting the conclusions of this article will be made available by the author, without undue reservation.

## Ethics statement

Ethical review and approval was not required for the study on human participants in accordance with the local legislation and institutional requirements. The patients/participants provided their written informed consent to participate in this study.

## Author contributions

SK built the study framework, analyzed the data, and wrote the manuscript.

## Funding

This study was supported by “Cornell College” in the United States.

## Conflict of interest

The author declares that the research was conducted in the absence of any commercial or financial relationships that could be construed as a potential conflict of interest.

## Publisher’s note

All claims expressed in this article are solely those of the authors and do not necessarily represent those of their affiliated organizations, or those of the publisher, the editors and the reviewers. Any product that may be evaluated in this article, or claim that may be made by its manufacturer, is not guaranteed or endorsed by the publisher.
